# Physico-Chemical and Metagenomic Profile Analyses of Animal Manures Routinely Used as Inocula in Anaerobic Digestion for Biogas Production

**DOI:** 10.3390/microorganisms10040671

**Published:** 2022-03-22

**Authors:** Asheal Mutungwazi, Grace N. Ijoma, Henry J. O. Ogola, Tonderayi S. Matambo

**Affiliations:** 1 Institute for the Development of Energy for African Sustainability (IDEAS), College of Science, Engineering and Technology, University of South Africa (UNISA), 28 Pioneer Ave, Cnr Christiaan De Wet & Pioneer Rds., Florida Park, Roodepoort, Johannesburg 1709, South Africa; amutungwazi@gmail.com (A.M.); nkechiijoma@gmail.com (G.N.I.); 2 Centre for Research, Innovation and Technology, Jaramogi Oginga Odinga University of Science and Technology, Bondo P.O. Box 210-40601, Kenya; henryoogola@gmail.com

**Keywords:** autochthonous, microbiome, feed composition, nutrient utilisation, gastrointestinal tract

## Abstract

Anaerobic digestion (AD) of organic waste is considered a sustainable solution to energy shortage and waste management challenges. The process is facilitated by complex communities of micro-organisms, yet most wastes do not have these and thus need microbial inoculation using animal manures to initiate the process. However, the degradation efficiency and methane yield achieved in using different inocula vary due to their different microbial diversities. This study used metagenomics tools to compare the autochthonous microbial composition of cow, pig, chicken, and horse manures commonly used for biogas production. Cows exhibited the highest carbon utilisation (>30%) and showed a carbon to nitrogen ratio (C/N) favourable for microbial growth. Pigs showed the least nitrogen utilisation (<3%) which explains their low C/N whilst horses showed the highest nitrogen utilisation (>40%), which explains its high C/N above the optimal range of 20–30 for efficient AD. Manures from animals with similar gastrointestinal tract (GIT) physiologies were observed to largely harbour similar microbial communities. Conversely, some samples from animals with different GITs also shared common microbial communities plausibly because of similar diets and rearing conditions. Insights from this study will lay a foundation upon which in-depth studies of AD metabolic pathways and strategies to boost methane production through efficient catalysis can be derived.

## 1. Introduction

Micro-organisms are ubiquitous in the natural environment [1]. Knowledge of the microbial diversity within an environment is important for the conservation of the various biological ecosystems. Such understanding is pertinent when we apply the epistemology derived in the operations of artificial biological reactors. Moreover, to improve efficiencies in scale-up, a typical biotechnological process such as an anaerobic digester should apply a clear understanding of microbial diversity, behaviour, and successional patterns observed during the degradation of organic wastes to efficiently produce bio-methane fuel [2,3,4]. The AD process is understood to have four syntrophic stages: hydrolysis, acidogenesis, acetogenesis, and methanogenesis but is, however, a very complex process that significantly depends on the interaction of microorganisms participating in a myriad of functions within both sequential and simultaneous pathways that can be summed in the above-mentioned stages. A good understanding of the food web, microbial abundance, interaction of microorganisms in the bioreactor, the presence and absence of inhibitory and promoting factors as well as microbial responses under certain conditions is therefore fundamental in methane yield optimisation [4]. Each step within these sequential and simultaneous degradation processes is dependent on the presence of the responsible microorganisms and the consequential production of the enzymes at concentrations adequate to achieve the intermediate products for the forward biochemical reactions and metabolite production. If the ratios of these enzymes (microorganisms) are reduced within the microbial consortia due to factors such as changing substrate compositions and concentrations, the outcome of the anaerobic digestion process and the ultimate methane yield will be compromised [5,6]. Archetypal designs of the anaerobic digestion (AD) for biogas production utilises various organic wastes such as grass-cuttings, kitchen wastes, agricultural wastes, and even municipal organic sewage with the inclusion of faecal matter as inocula obtained from various animals. The premise of the application of these types of inocula is that they provide micro-organisms (bacteria, fungi, and protozoa) that secrete enzymes that are active in the degradation of lignin and lignocellulosic substrates as well as methanogens needed for bio-methane production. Different animals produce manures with different consistencies, quantities and subsequently affect the qualities of bio-methane, due to the varied organic composition of the substrate ingested. Moreover, the diversity of micro-organisms present in the inoculum and substrate introduced into the digester are significantly affected by the physical and chemical operational conditions used during the AD process [7,8]. However, there is a dearth of knowledge of the diversity and functional characteristics of micro-organisms natively present in these manures that serve the dual roles of substrate and inoculum or just the latter [4,9]. This limited knowledge of microbial consortia function and interaction in ADs can be identified as a significant factor to systems failure and sometimes inefficient production of biogas, even in some of the countries considered to be advanced and at the forefront of the AD technology [10].

Being cognisant of the knowledge gap highlighted above, it is prudent that the microbial diversity of animal manures fed into AD systems be determined as this would provide, significant insights into the interspecific interactions of the microbial population dynamics as well as the identification of the possible functional roles of these organisms towards biogas production. In the past, there existed a limitation to microbial consortia characterisation, however, advances in molecular biology and omics technologies, provide opportunities to expand our knowledge and increase applications and process optimisation. Technological advancements in metagenomics and next-generation sequencing (NGS), a high-throughput process that sequences hundreds to thousands of genes at a time and has the ability to detect novel or rare variants with deep sequencing, have bridged the gap created by the conventional single-cell culturing methods [11]. NGS tools are capable of determining the composition of microbial ‘communities’ in a single run and they can also accurately establish the association between a host and its microbiome [12]. Consequently, wastes such as cow dung [13,14,15,16,17,18], horse manure [19,20], chicken manure [18,21], and pig manure [15,22,23], that are commonly used as both substrate and inoculum digested for methane production, can now be analysed for microbiome affordably and efficiently using NGS platforms. The microbial diversity of the aforementioned animal manures varies and is shaped by several complex factors which include diet [24,25], digestive system [26], and environment [25,27,28]. Of special interest in this study, are the five dietary elements: Carbon (C), Hydrogen (H), Oxygen (O), Nitrogen (N) and Sulphur (S). Thus, the aim of this study is to determine by comparison, the bio diversities of cow, pig, horse, and chicken manure with respect to feeding composition, gastrointestinal tracts physiology, and location, and the potential effects of these factors on biogas yield during anaerobic digestion.

## 2. Materials and Methods

### 2.1. Animal Feed and Manure Sample Collection

Seven locations were identified in and around Johannesburg in South Africa as shown in Table 1, for the collection of feeds and triplicate samples of fresh manure from cows, chickens, horses, and pigs. All samples were collected in the summer season (February to March) within an interval of three days and processed for analysis as a batch. To ensure biological replicates, samples were collected from varied locations. Upon collection, all the samples were put in sterile plastics and stored in a portable icebox in which they were then transferred to the laboratory at the University of South Africa (UNISA), Johannesburg for physico-chemical and microbiome analyses.

### 2.2. Physico-Chemical Analysis of Animal Feeds and Manures

Fresh samples of the animal feed and manures were finely ground using a pre-sterilized coffee grinder (SCG-250) to a particle size diameter of approximately 2 mm. The samples were then air-dried for 24 h in preparation for elemental analysis. The elemental analysis to determine the carbon (C), hydrogen (H), nitrogen (N), sulphur (S), and oxygen (O) composition in the samples followed the protocol as described by Krotz and Giazzi [29]. In this protocol, a Thermofisher FLASH 2000 organic elemental analyser is used. Samples to be analysed are weighed in a tin capsule and introduced into a combustion reactor via the Thermo Scientific™ MAS™ 200R auto-sampler together with a determined proportional amount of oxygen. After combustion, the resultant gases are carried by helium into a gas chromatography (GC) column which separates them into their elemental constituents for detection by a thermal conductivity detector (TCD) to generate a report using the Thermo Scientific™ Eager Xperience data handling software.

### 2.3. Microbiome Characterisation

#### DNA Extraction and Bioinformatics Analysis

Genomic DNA was extracted from the manure samples using the DNeasy PowerSoil kit (QIAGEN, Hilden, Germany), following the manufacturer’s instructions. These samples were analysed in triplicates. The obtained genomic DNA was then amplified by polymerase chain reaction (PCR) using the universal bacterial primers, 27 F (5′-AGAGTTTGATCMTGGC-3′) and 518 R (5′-GTATTACCGCGGCTGCTGG-3′) targeting the conserved bacterial 16S rRNA gene as described by Selvarajan et al. [30]. A DNA Clean and Concentrator Kit (ZYMO RESEARCH, Irvin, USA) was then used to purify PCR products. Prior to the library preparation and sequencing process, the triplicate samples were pooled together before Illumina sequencing adapters and dual-index barcodes were added to the amplicon targets using a full complement of Nextera XT indices (Illumina, Inc., San Diego, CA, USA) through eight cycle PCR (95 °C for 3 min, 95 °C for 30 s, 55 °C for 30 s, and 72 °C for 30 s, with a final extension at 72 °C for 5 min, then cooling at 4 °C). AMPure XP beads were used again to clean the PCR product. A bioanalyzer DNA 1000 chip (Agilent, Santa Clara, CA, USA) was then used to validate the size of the fragments (~630 bp) before quantifying them in a fluorometric quantification method (Qubit, NY, USA) which uses dsDNA binding dyes. Dilutions were achieved on the quantified DNA using 10 mM Tris Buffer (pH 8.5). Five microlitre of diluted DNA was aliquoted from each library and mixed for pooling libraries. The pooled final DNA library (4 nM) was denatured and sequenced on an Illumina Miseq System using paired 300-bp reads to generate high-quality, full-length reads of the V3 and V4 regions in the College of Agricultural and Environmental Sciences (CAES) Department at UNISA, South Africa. From the Miseq, raw fastq sequence files were then obtained after trimming the adapters and primer sequences. Sequences derived were subjected to bioinformatics analyses. The fastq sequence files from the Miseq were inspected for quality using the FastQC software (v 0.11.7, Babraham Bioinformatics, Cambridge, UK). Using PANDAseq.37 on the QIIME2 platform, the forward and reverse reads were merged and then clustered into operational taxonomic units (OTUs) at a sequence similarity of 97% for species-level identification using the ‘pick_open.reference_otus.py’ script in QIIME, while aligning against the SILVA rRNA (release 132) database38 by using usearch61 and PyNAST aligner. The OTU table generated was exported to the R-studio for further statistical analyses. R packages: vegan, ape, labdsv, and ggplot were installed and used for statistical analyses and plotting [31].

## 3. Results

### 3.1. Elemental Composition of Animal Feeds and Manures

Table 2 below shows the utilisation rates of C, H, N, and S nutrient elements by the animals under investigation. These utilisation rates were derived using the apparent digestibility method, from Table A1 and Table A2 (in Appendix A) which show the elemental analysis results for the animal feeds and manures, respectively. The elemental analysis results in Table 3, show that carbon was a major component of all the animal feeds ranging between 30 and 48%. Hydrogen percentages are ranging between 4 and 7% (6–7 times less than carbon) and nitrogen ranged between 0.9 and 4.5%. Sulphur has the least quantities ranging between 0 and 2.3%.

From Table 2, cows exhibited the highest carbon utilisation (above 30%) followed by horses then pigs, and lastly, chickens (below 8%). Notably, pigs proved to have the least nitrogen (below 3%) and highest sulphur (above 50%) utilisation whilst horses were shown to use nitrogen nutrients the most (above 40%). This gives rise to the high concentration of carbon and nitrogen in horse and pig manures respectively as shown in Table A2 (see Appendix A). Chickens exhibited the highest utilisation rate of hydrogen, followed by cows, then horses, and lastly, pigs. Generally, all the animal species significantly utilise sulphur (above 13%).

### 3.2. Taxonomic Diversity of Bacterial Communities in Manure

The filtering out of low-quality and chimeric sequences yielded a total of 262,199 quality reads from the 12 animal manure samples. These sequences were employed for the downstream analysis. H2 had the highest number of valid reads (71,805), whereas, H1 had the least number of valid reads (5152). Although, according to Ni et al. [32], this quantity, even though minimal, is acceptable for credible microbiome analysis. Overall, 9355 OTUs were elucidated across all the samples based on the Silva rRNA gene database at the cut-off level of 97%. Among the twelve animal manure samples, pig manure P1 (1628 OTUs) had the highest number of unique OTUs, while the lowest number of OTUs were recorded for chicken manure F2 (103 OTUs). The calculated alpha diversity indices including the six are presented in Table 3.

The Chao_1 and ACE indices were used to estimate the expected OTU richness based on the observed OTUs, and they both showed the lowest number of OTU richness in chicken manure F2 (188.55) and the highest in pig manure P1 sample (1643.50). The Shannon-diversity index which places a greater weight on species richness showed that the highest diversity was in cow manure C3 (6.07) while the lowest was in horse manure H2 (1.00). There was no significant difference between the bacterial diversity (Shannon_H) and the bacterial species richness (Chao_1) across all the samples. The Simpson_1-D index showed that C1 had the highest species evenness (0.94) while H2 and P3 had the lowest species evenness (0.30).

### 3.3. Phylum and Family Diversity

**Figure 1:**
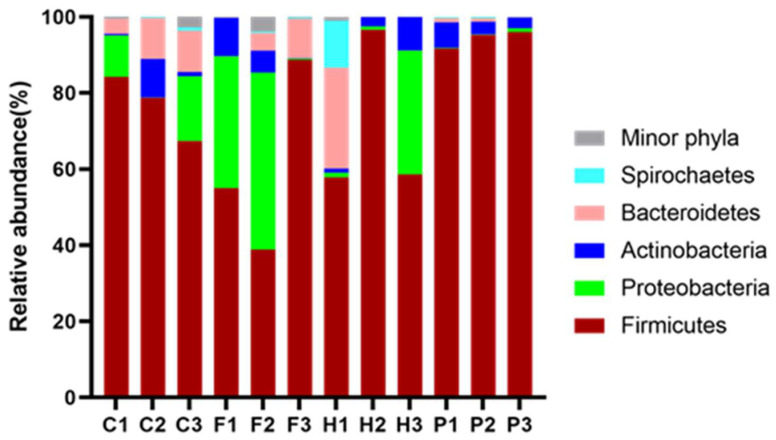
Relative Abundance of Sequences Representing Phyla Constituting Bacterial Communities in Different Animal Manure Samples.

Phylogenetic classification revealed 20 bacterial phyla distributed across all manure samples. The five most dominant phyla were *Firmicutes* with a relative abundance ranging between 38.85% of the total 16S rRNA gene sequences recorded in F2 and 96.63% of those recorded in H2, *Proteobacteria* (0.08% in P3 to 46.54% in F2), *Bacteroidetes* (0.88% in P2 to 26.48% in H1), *Actinobacteria* (1.11% in H1 and 10.22% in C2) and *Spirochaetes* with an abundance of 12.38% in H1 only. Figure 1 shows the distribution of bacterial phyla in the animal manure samples.

**Figure 2:**
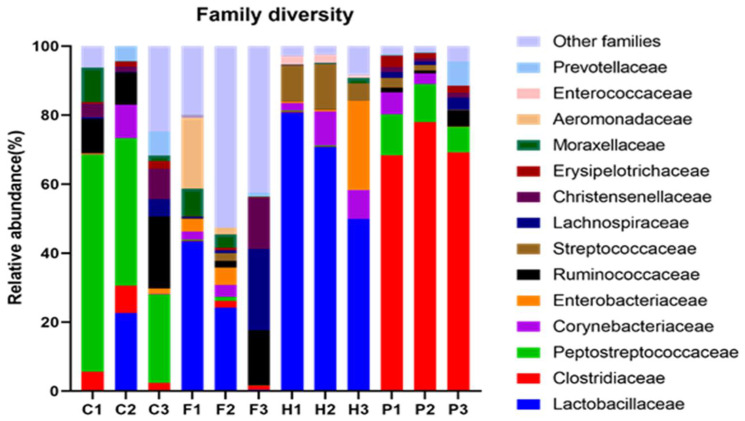
Relative Abundance of Sequences Representing Families Constituting Bacterial Communities in Different Animal Manure Samples.

Family level taxonomic classification of the bacterial communities in the animal manures showed that a total of 172 families were present across all manure samples. Figure 2 shows the distribution of the top 15 bacterial families in the animal manure samples.

The bacterial family *Peptostreptococcaceae* was remarkably abundant in all the cow manures ranging between 25.68% in C3 and 62.97% in C1, and notably present in all pig manures ranging within 7.36% in P3 and 11.85% in P1. The *Clostridiaceae* family was dominant in the three pig manures ranging between 68.22% in P1 and 77.85% in P2, and notably present in all cow manures at abundances ranging from as little as 2.31% in C3 to 7.90% in C2. *Lactobacillaceae* members were dominant in horse manures within the range of 49.82% in H3 and 80.82% in H1. The family was also present in poultry manures (24.23% in F2 and 43.46% in F1) and in cow manure, C2 at 22.65%. *Corynebacteriaceae* abundance in various manure samples ranged between 2.31% in F1 and 9.86% in H2. *Ruminococcacceae* was also abundant in cow manures ranging between 9.33% in C2 and 20.87% in C3 and notably present in F3 at a relative abundance of 15.97%. F3 contained 23.58% of *Lachnospiraceae* and 14.79% of *Christensenellaceae*. *Streptococcaceae* were identified in all horse manures within the range of 5.00% in H3 and 13.08% in H2. *Aeromonadaceae* and *Enterobacteriaceae* were remarkably present in F1 (20.40%) and H3 (25.81%) respectively. *Moraxellaceae* had a relative abundance of 1.60% in H3 and 9.95% in C1. Members of the family *Prevotellaceae* were also notably present in C2, C3, and P3.

### 3.4. Genus Diversity in Manure Samples

**Figure 3:**
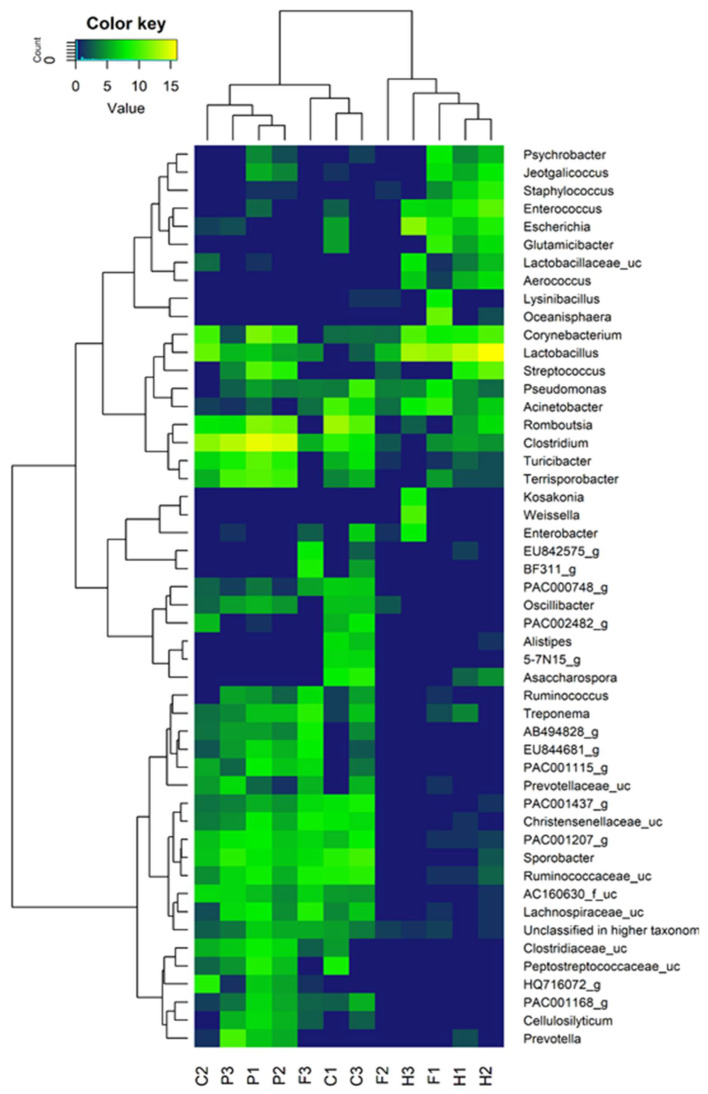
Genus Level Distribution of Bacterial Communities in Different Animal Manure Samples.

The genera identified in the triplicate manure samples from each animal species were analysed for similarities and differences. Genera found to be commonly present in all the three samples were identified as well as those uniquely identified in each sample and not in other samples. The prevalent genera unique to C1 were *Glutamicibacter*, *Enterococcus*, and *Jeotgalicoccus* while those prevalent in C2 only were *Prevotella* and *Lactobacillaceae_uc* and those uniquely identified in C3 were *Cellulosilyticum, Enterobacter, Lysinibacillus*, and *Psychrobacter*. Common genera identified in all cow manures were *Lachnospiracaeae_uc, Ruminoccocaceae_uc, Sporobacter, Christensenellaceae_uc, Treponema, Oscillibacter, Terrisporobacter, Turicibacter, Clostridium, Romboutsia, Acinetobacter,* and *Corynebacterium.* For chicken manures, the genera common in all the manures were *Clostridium, Acinetobacter, Pseudomonas,* and *Lactobacillus*. Prevalently unique to F1 were, *Oceanisphaera, Glutamicibacter, Escherichia, Enterococcus,* and *Jeotgalicoccus*. *Oscillibacter* and *Streptococcus* were only identified in F2 while *Christensenellaceae_uc* and *Sporobacter* were only identified in F3. In horse manures, the genera *Romboutsia, Acinetobacter, Pseudomonas, Lactobacillus, Corynebacterium, Aerococcus, Lactobacillaceae_uc, Escherichia,* and *Enterococcus* were commonly identified. However, H1 had *Prevotella, Christensenellaceae_uc,* and *Treponema* prevalently unique to it while H2 had *Lachnospiracaeae_uc, Sporobacter, Alistipes,* and *Oceanisphaera* prevalent only in it, and H3 uniquely harboured *Enterobacter, Weissella,* and *Kosakonia*. The genera *Prevotella, Cellulosilyticum, Peptostreptococaceae_uc, Clostridiaceae_uc, Lachnospiracaeae_uc, Ruminoccocaceae_uc, Sporobacter, Christensenellaceae_uc, Treponema, Ruminococcus, Oscillibacter, Terrisporobacter, Turicibacter, Clostridium, Romboutsia, Pseudomonas, Streptococcus, Lactobacillus,* and *Corynebacterium* were commonly identified in all pig manure samples. *Acinetobacter, Lactobacillaceae_uc,* and *Enterococcus* were however only identified in P1 while *Enterobacter* and *Escherichia* were prevalently identified uniquely in P3. Figure 3 shows the genus level distribution of bacterial communities in different animal manure samples.

## 4. Discussion

### 4.1. Taxonomical Similarities and Differences in Biological Replicates of Manures from the Same Animal Species

Remarkable similarities in the genera among biological replicates of manure samples were observed for the same species collected from three different sources and locations. Moreover, as was expected there were microorganisms that were commonly found in all manure samples of the various animals’ manure samples. However, some notable differences existed in each of the samples, moreover, for P2 (pig manure from Bosheuvel Country Estates), there was at least a genus uniquely identified that was absent in all other samples. A likely indication that the presence of the microorganisms in these samples might be a consequence of the environment. From the cow manure biological replicates, it is not very clear why the genera *Glutamicibacter, Enterococcus,* and *Jeotgalicoccus* were only identified in C1 and not in the other cow manure samples but a plausible explanation could be that these genera are ubiquitous and can be found in certain soils, water, air, etc., [33,34].

In any case, *Prevotella* was identified in C2, most likely the result of the free-range feeding habits observed at Kate’s Farm (C2) with the implication that these cows are less likely to suffer from bovine rumen acidosis which often is a common occurrence with cattle that are overfed particularly with significant quantities of rapidly digestible carbohydrates found in feed preparations due to economic considerations [35]. The growth of *Prevotella* is linked to inhibition of acidic pH conditions such as those created by short-chain fatty acids when the cow host suffers rumen acidosis [36]. This explanation is supported by observations made by Iljazovic et al. [37], in their study, with a unique strain of this bacteria, *P. intestinalis,* that demonstrated the ability to alter the composition of the animal gut ecosystem resulting in a reduction of these short-chain fatty acids, specifically acetate, and consequently decreasing intestinal Interleukin-18 (IL-18) levels thus preventing bovine rumen acidosis [38]. It is likely that the identification of *Lactobacillaceae_uc*, only in C2 as well, is because of its requirements of complex media for growth, most likely fulfilled in the free-range feeding in this farm. Moreover, the fastidious pH requirement of above 6 for these bacteria, is often difficult to maintain in the GITs of cows ‘overfed’ on supplementary feeds which expose them to rumen acidosis (C1 and C3) and consequently lower pH.

*Cellulosilyticum* was uniquely identified in C3 (Bosheuvel Country Estates) most plausibly because the cows at this site subsisted on a plant-based diet comprising mixed vegetables as indicated in Table 2. This genus utilises cellobiose, cellulose, xylose, maltose, and xylan from plant material as carbon and energy sources to produce acetic acid, formic acid, carbon dioxide, and trace amounts of lactic acid, succinic acid, and ethanol during fermentation [39]. Similarly, the presence of *Enterobacter* in the same samples is possibly the result of ingesting fresh vegetable matter carrying the genus at any point of its nitrogen fixation cycle as postulated by Rogers [40]. *Glutamicibacter, Enterococcus,* and *Jeotgalicoccus* were not only identified in the cow manure from Kate’s farm but also in the chicken manure (F1) from the same farm and not in any other chicken manure from other sources. The presence of these organisms in these two farm animals implies a likely association with the environment (location). The chickens in Kate’s farm are likely to be cultivated roadrunners meaning that their diet consisted of worms, bugs, insects, and undigested foodstuffs such as grains from the fresh or dry dung of cows thus simultaneously ingesting the micro-organisms commonly present in the rumen and other parts of the digestive tract of cows as indicated by Cai et al. [41], Difford et al. [42], as well as Pan and Yu [43]. A very old study done by Hammond [44], suggested that cow manure significantly improves growth in chicks if added to a riboflavin deficient diet. *Christensenellaceae_uc* were only identified in F3 possibly because some of its species produce short-chain fatty acids acetate and butyrate, and are saccharolytic, with the ability to utilize glucose, rhamnose, arabinose, salicin, mannose, and xylose as demanded by the plant-based diet (mixed vegetables) of the chickens at Bosheuvel Country Portion. However, H1 had *Prevotella, Christensenellaceae_uc,* and *Treponema* prevalently unique to it hilee H2 had *Lachnospiracaeae_uc, Sporobacter, Alistipes,* and *Oceanisphaera* prevalent only in it and H3 uniquely harboured *Enterobacter, Weissella,* and *Kosakonia. Acinetobacter, Lactobacillaceae_uc,* and *Enterococcus* were however only identified in P1 while *Enterobacter* and *Escherichia* were prevalently identified uniquely in P3.

### 4.2. Feed Composition Influence on Faecal Microbiota of Cow, Chicken, Horse, and Pigs

The elemental analysis results in Table 2 show that carbon was a major component of all the animal feeds ranging between 30 and 48%. Hydrogen percentages are ranging between 4 and 7% (6–7 times less than carbon) and nitrogen ranged between 0.9 and 4.5%. Sulphur has the least quantities ranging between 0 and 2.3%. Carbon quantities generally decreased by about 10–12% in C1 and C3, between 1 and 4% in H1 and H3, about 4% in F1, F2, and F3, and between 1 and 3% in P1 and P2, signifying the higher consumption of carbon in cows compared to other animal species. Remarkably, the carbon quantity in P3 manure increased by about 2%. Nitrogen in cow manures increased by a percentage between 1 and 4% from the quantities measured in the feeds.

There is increasing recognition of the role of an animal’s diet with reference to feeding composition in modulating the biodiversity (microbial fauna) and metabolic activity of the animals’ gut microbiota, which in turn can impact health. Macronutrients play a major role in shaping the composition and activity of complex microbial populations [45]. The digestive system uses enzymes and microbial fermentation to break down feed into small molecules which can then be absorbed into the body. It also serves as a barrier against antigens and pathogens since it is the largest interface between the host and the environment. The diet can control the immune function of the digestive system by influencing the elemental composition and the metabolic activity of the GIT microbiota and enhancing the production of antimicrobial peptides that interfere with the growth and the adhesion of pathogens to the intestinal mucosa. The intestinal microbiota in turn contributes to the protective functions of production of antimicrobial factors, pathogen displacement, fermentation of non-digestible dietary residue, nutrient competition, synthesis of vitamins, and metabolism of xenobiotics [46,47]. Observations made in this study reaffirm the pivotal importance of the carbon element as a major component of all the animal feeds (see Table 2); further noting that it is primarily found in all food classes, especially in carbohydrates, which is the predominant food required for metabolic energy production needed for activities such as blood and gas circulation, breathing, digestion, regulation of body temperature, tissue repair, activities such as grazing, growth, walking, milk production and maintaining pregnancy [48,49]. This higher requirement of carbon was observed in feed preparations of the large animals including cows, horses, and pigs, respectively. Additionally, the abundance of carbon in horse manure could indicate the presence of some additional carbon bedding mixed with the horse manure in the feeding pen [50,51].

Even though energy is the main driving force of metabolism, other elements such as hydrogen, oxygen, nitrogen, and sulphur are also important components in the complex structures of all food classes including proteins, fats, and oils as well as vitamins. These food classes are also crucial to metabolic functioning. The ratios of these elements vary, and the vast body of knowledge amassed in animal nutrition has made it possible to develop feeds that take this understanding into consideration in its preparation [52,53,54,55]. This is important to ensure efficient use of these nutrients by these animals as well as guaranteed profitability in meat production. For example, nitrogen elemental composition in feed preparations for cows was generally higher than that in feeds for other farm animals represented in this study because it is likely, considerations were given to the pivotal role of the symbiotic microbial population resident in the GIT, that participate in the lignocellulosic carbohydrate digestion. The health and protein requirement of these microorganisms is very important in ensuring a positive symbiotic relationship with the host animal [56]. Ruminants, therefore, need more protein than animals with other digestive systems. Both ruminants (cows) and pseudo-ruminants (horses) have foregut and caecum capacities in the range of 150–180 L and 100–140 L, respectively. The foregut and caecum contain microbiota that break the cellulose and lignin feeds to expose the carbon in them [46]. Nitrogen in cow manures increased from the quantities measured in the feeds plausibly because the excess protein went through deamination and was passed out as urea in urine as well as in mixtures with cow dung [56,57].

Although sulphur is an important dietary requirement, there is a strict consideration to ensure it is supplied in very minute quantities since a severe build-up of this element can result in neurological changes such as coma, blindness, and recumbence, therefore, only limited amounts are included in feeds. This is supported by Erickson and Kalscheur [58] who recommended feeding a 0.5% sulphur diet on a dry mass basis to avoid toxic levels and cases of dietary cation-anion difference (DCAD) in dairy cows. The present work focused on the influence of feed composition on GIT and consequently faecal microbiota, with particular emphasis on 16S targeted investigation of bacteria in manure samples as the basis of exploratory investigation of biodiversity of the different environments and compositional variations represented within each sample. This is because bacteria are by far the most dominant (>97%) microbiota in the GITs of farm animals while viruses, fungi, and archaea are a rare biosphere comprising less than 3% of the microbiome [59]. The farm animals selected in this work, i.e., cow (ruminant), horse (pseudo-ruminant), pig (monogastric), and chicken (avian), were selected to purposefully represent the various GIT mechanisms commonly found in farm animals and possibly determine if this may also contribute a physiological influence on the type of microbial communities present within these host animals. Moreover, biological replicates (triplicates from different locations) of samples were collected to ensure the reproducibility of findings. The 16S rRNA sequencing approach was chosen for the characterization of the bacterial communities and taxonomical identification owing to the necessary economic consideration involved with the large sampling bacterial cohort [60].

This study identified the bacterial consortia in the different manures beginning at phylum levels for all farm animals represented (see Figure 1). *Firmicutes* were identified as the most highly abundant phylum. It is not surprising, as they are robust endospore producers, this trait confers them with abilities to resist desiccation and survive extreme conditions such as changing pH, oligotrophic and metal-rich environments [61]. Although the ubiquity of *Proteobacteria* is notable in most environments, due in part to their motility and ability to facilitate colonization and biofilm formation [62], it is remarkable that they were not observed in samples of the pig manure from all locations, and it is worth further investigation as to the reasons for this notable absence. *Actinobacteria* was also found in nearly all samples at varying quantities. The phylum *Actinobacteria* is common in soils, playing an important role in the decomposition of organic materials such as polysaccharides, cellulose, protein fats, organic acids, and chitin. Although not found in large quantities in most samples, most likely due to their high sensitivity to acid and low pH [63], their presence in these samples, is possible contamination from the soil medium mixed with the animal droppings [64,65].

*Bacteroidetes* were identified in both cow and horse manure. These groups of bacteria mostly inhabit the distal gut of these farm animals, where they are active in providing the host with energy obtained from the diet through the fermentation of indigestible polysaccharides. This activity produces short-chain fatty acids (SCFAs) that can supply up to 10% of daily calories when the diet is rich in fibre [66,67]. It is most likely that the presence of *Bacteroidetes,* observed in chicken manures might be due to the chickens picking some bugs, worms, and undigested feed from the cow manures. Most *Spirochaetes* are free-living and anaerobic, other species are important symbionts in the stomachs of cows and other ruminants [68]. Their presence in horse manure (H1) could be due to the anaerobic conditions prevailing in the caecum.

### 4.3. Feed versus Manure Compositions, and Their Likely Influence on Biogas Production

The nutrient composition (quality and biological availability) of a given animal feed determines how much of the feed must be fed to the animal to meet its nutritional requirements and how much will eventually be excreted in the manure. Improving the digestibility of the nutrients can also reduce the loss of the nutrients through excretion. According to VanHorn [69] and Manitoba [70], livestock typically excrete 50 to 90% of the nutrients they are fed, depending on the animal species, stage of growth, and the ration provided (feed source and supplements). Fully-grown animals that are not gaining weight, gestating or producing milk or eggs, however, excrete almost all of the nutrients they are fed. Carbon and nitrogen are the most important of the many elements required for microbial decomposition of organic matter to produce compost as carbon makes up about 50 percent of the mass of the cell of microorganisms, provides an energy source while nitrogen is a key component of proteins, and is essential for microbial growth [71]. In AD systems, the microbial uptake of livestock’s carbon and nitrogen nutrient utilisation is a function of the quantitative relationship between the two amounts, expressed as a ratio (C/N) in the livestock’s manure when it is used as a substrate, it is reflected in the degradation process efficiency. A balance of these elements should be maintained to keep the microorganisms at their optimal operating condition and a C/N ratio of 25:1 to 30:1 has been recommended [72,73,74]. C/N lower than this results in excess nitrogen and the production of ammonia while a higher C/N deprives the microorganism of enough nitrogen to support sufficient microbial growth and the biomass population would decrease, thereby slowing the AD process down. The variation of the C/N values also affects the pH of a slurry to the detriment of some methanogens, leading to digester souring [75]. Pig, chicken, horse, and cow manure samples in this study had C/N ratios of 6.3, 9.0, 34.7, and 22.3 respectively. These values correspond with C/N ratio findings in the literature of 6 for pig manure [76,77], 3–10 for poultry manure [77], 20–50 for horse manure [76], and 20–25 for cow manure [68,69], respectively. It, therefore, follows that cow manure would provide the most comfortable condition for the growth and population of microorganisms whilst horse manure, being highly carbonic would require co-digestion with a high nitrogen content substrate such as pig and chicken manure.

Sulphur is needed for the growth of bacteria and synthesis of cell materials and sulphur-containing cofactors involved in cellulose degradation and methanogenesis. In a study on the effect of sulphur-containing compounds on AD of cellulose to methane by mixed cultures obtained from sewage sludge, Khan and Trottier [78] observed that a sulphur source of about a 0.85 mM concentration is essential for the degradation of cellulose to CH_4_. However, in their study, a concentration of 9 mM inhibited both cellulose degradation and methane formation, and this inhibition increased in the order thiosulfate < sulphite < sulphide < H_2_S. In this study, chickens showed the least apparent digestibility of sulphur i.e., they were proven to have the least sulphur utilisation, followed by horses. This is a plausible explanation for the poor degradation of cellulose in horses and low methane production from the manures of horses and poultry.

The formation of sugars, proteins, starch, and fats, all of which form the diet of the animals, requires hydrogen and oxygen. In the digestive system of ruminant animals, hydrogen in all its forms plays the role of central regulator. The redox potential of the rumen and the possible extent of oxidation of feedstuffs is directly determined by the balance of the concentrations of dissolved hydrogen gas (H_2_) and the hydrogen ion (H^+^). Hydrogen for methane synthesis occurs in three key states in the rumen; hydrogen gas (H_2_), reduced cofactors such as nicotinamide adenine dinucleotide (NADH) and nicotinamide adenine dinucleotide phosphate (NADPH), and free protons [79]. Essentially H^+^ ions are fundamental to the several metabolic processes in all living things including all livestock.

Microbial characterisation at the family level, identified about 15 major families. Bacterial families in the *Firmicutes* phylum were observed to be *Peptostreptococcaceae, Clostridiaceae, Lactobacillaceae, Ruminococcaceae, Lachnospiraceae, Streptococcaceae,* and *Christensenellaceae. Peptostreptococcacceae*. Members are predominantly anaerobes with a fermentative type of metabolism. Their prevalence in most manures is the reason for their favoured application in anaerobic digesters and for biogas production. Indeed, there are several studies that show the feasibility of biogas production from farm animal manures, an example is the study by Salam et al. [80], in which they measured biogas yield of about 27.3 L/kg of cow dung under lab-scale mesophilic conditions, Ulusoy et al. [81], also measured about 8.58 million m^3^ of biogas per year after processing approximately 110 thousand tons of chicken manure. Pertinently the study by Ulusoy et al. demonstrated the conversion of the biogas yield and the application to the generation of 17 GWh/year of electricity and 16 GWh/year of thermal energy. Okewale and Adesina [82], measured biogas with about 64% methane yield in their co-digestion studies of pig manure, poultry manure, and water hyacinth while, Agayev and Ugurlu [83], measured biogas yields of 339 mL/gVS, 374 mL/gVS, 370 mL/gVS, 381 mL/gVS for 0,5 %, 1%,2% and 4% TS horse manure contents respectively in batch and semi-continuous digesters under mesophilic conditions over a retention period of 35 days. The *Clostridiaceae* family was dominant in pigs and plausibly, its presence there could be explained by the monogastric GIT nature of pigs which has high protein and fat digestibility well adapted to this bacterial family. This notion is inferred from the findings of Bermingham et al. [84], in their studies on dogs which showed clear associations of *Clostridiaceae*, *Erysipelotrichaceae,* and *Bacteroidaceae* with protein and fat digestibility in the dog GIT. *Lactobacillaceae* dominated the horse manure microbiota. The horse feeds analysed in this work had whey as a constituent ingredient and hence the prevalence of *Lactobacillaceae* in the manures [85]. Whey supplies the horse with essential amino acids such as leucine, lysine, valine, and isoleucine which support the development of lean muscle mass for the enhancement of horse racing efficacy [86,87,88].

Studies by Mehmood et al. [89], highlighted that a diet rich in whey protein was observed to increase the population of the *Lactobacillaceae* family while decreasing the number of *Clostridiaceae*. *Lachnospiraceae* and *Ruminococcaceae* are notably identifiable in all manures from the active plant ingesting cows [90]. Members of the *Ruminococcaceae* and *Lachnospiraceae* can degrade cellulose and hemicellulose in gut environments and, hence, they can decompose components of plant material and substrates that would otherwise be indigestible by the host. Products of this decomposition are then fermented and converted into short-chain fatty acids (mainly butyrate, acetate, and propionate) that can be assimilated and used for energy by the host. *Christensenellaceae,* a recently described bacterial family that shows compelling associations with host health and is highly heritable [91,92], was also present in various animal species manure samples but with no unique prevalence. The family *Streptococcaceae* was notably visible in horse manure samples. Most of the species in the family are however known to be facultative anaerobes and only a very few are obligate anaerobes [93]. In this family is the genus *Streptococcus equinus*, an antibiotic-resistant commensal bacteria in horses [94], which intracellularly ferments alpha bonds of large, alpha-linked polysaccharides, such as starch and glycogen to yield glucose and maltose as a result of the enzymes α-amylase and amylomaltase [95].

*Corynebacteriaceae* family of bacteria belongs to the phylum *Actinobacteria* [96]. Members of the *Corynebacteriaceae* family such as *Corynebacterium* as are capable of producing biosurfactants that are implicated in foaming occurrence within AD systems thus significantly reducing bio-methanation efficacy [10]. According to Li Yang [97], *Enterobacter* and *Clostridium* are the major microorganisms that produce hydrogen from food waste, e.g., during the breakdown of catabolized sugar (but not protein or fat), as the carbon source but however, carbohydrate-based waste has higher H_2_ production potential than that of fat-based and protein-based waste. It would thus follow that the family *Enterobacteriaceae* which belongs to the *Proteobacteria* phylum was notably identified in chicken and some horse manures probably owing to their diet being rich in significant amounts of catabolized sugars. The *Prevotellaceae* family is from the *Bacteroidetes* phylum and the genus *Prevotella* also commonly found in the human gastrointestinal microbiota, has culturable species that are among the most numerous microbes prevalent in the hindgut and rumen of sheep and cattle helping in the breakdown of carbohydrates and proteins [98]. The current work agrees with Jang et al. [98], as the *Prevotellaceae* members in this work were identified in cow manures. Some were however also identified in horse manures, plausibly due to the caecum conditions that mimic the rumen and hindgut environment. Manure samples from the four animal species had many bacterial genera in common. Of interest in this work were the genera relevant to biogas production through the anaerobic digestion process. These were selected and tabulated in Table A3 and Table A4, Appendix B.

An analysis of the dominant AD-related bacterial diversities in the manures of each animal species revealed that the dominant AD-related genera among the various manures were taxonomically similar. This suggests that basically, all the analysed manures can be considered for AD process inoculation especially considering the discussed issues to do with similar location and feed concentrations (see Table 1) which give rise to microbial taxonomy similarities in some of the manures regardless of the different animal species producing them. The animals’ GITs however differ, and this plausibly results in some microorganisms being uniquely identified in certain GITs. Identified cases are the genera, *Weissella* and *Kosakonia* which were unique to horse manure, and *Peptostreptococaceae_uc* and *Terrisporobacter* unique to pig manure. This may be because *Weissella* have complex nutritional requirements and they inhabit nutrient-rich environments such as the human GIT fed with raw milk, fish, blood, meat, soil, etc., therefore since some of the horses studied had a diet comprising of whey and other special feeds containing protein and other nutritional requirements as shown in Table 2, the prevalence of *Weissella* was observed [99,100]. *Weissella* plays an important role in food fermentations based on vegetables or meat as substrate as well as in fermentation processes such as the production of silage. Application of horse manure to the AD of kitchen waste or any other waste containing vegetable and protein matter on this basis would possibly increase the biogas production process efficiency. Besides the assertion that *Kosakonia* is typically found in natural environments, water, soil, and sewage [101,102], its unique presence in horse manures needs further investigation, although it is likely a consequence of interactions by the animals with any one of the three environments mentioned. *Peptostreptococcaceae*_*uc* produces acid from glucose and other carbohydrates and grows very slowly. This quality favours AD for biogas production as the genus-group would last longer during the process, performing the desired bio-catalysis role. The genus *P. anaerobius* is known to be biochemically inactive and does not ferment carbohydrates [103]. It is an acid-tolerant genus and thrives comfortably in the monogastric GIT of a pig which secretes enzymes to break down food into smaller particles and have additional acidic gastric juices produced by the salivary glands, liver, and pancreas to assist with the digestion of food. The secreted acid can result in a pH as low as 1.5 to 2.5 which makes pigs better adapted to rations that are high in grain concentrates as compared to forage [104]. This could explain the prevalence of the acid tolerant *Peptostreptococcaceae*_*uc.* This makes pig manure a good AD process stabiliser as it contains microorganisms that can tolerate the acidic conditions during acidogenesis and continue the degradation process, preventing acid inhibition and process souring from occurring. The chicken manure samples had the least dominant AD micro-organisms plausibly because they have an avian GIT length which is much shorter, relative to body length, than that of mammalian animals and as such, the retention time of food in the digestive system is short (less than 3.5 h on average) [43]. This short retention time only favours bacteria that are fast-growing unlike those genera found in the long GITs of ruminants and pseudo-ruminants. Chicken manure is therefore the least likely to provide meaningful AD process inoculation since its microbiome may not last long enough to complete the retention time/s of the metabolic pathways involved in AD.

## 5. Conclusions

This work highlighted the influence of factors that include, the gastrointestinal tract, diet, and location of an animal on its faecal microbiota (manure). Biological replicates (triplicates) of manure samples collected from different sources showed similar genera commonly identified amongst them all to a larger extent, implying their basic potential use as inocula for AD systems. A few genera unique to specific sources were however identified as well. Pertinent differences in physiological characteristics of the GITs (monogastric, avian, ruminant, or pseudo-ruminant) appear to contribute to the variations in microbial diversity and are likely to influence the extent to which the microorganisms thrive in the different GIT environments and the organisms that are thus predominant in the various livestock’s manure. On this basis, the manures’ application as inocula in AD processes can potentially bring about different effects. A typical example was the observation of some bacterial genera only in horse manures and some only in pigs due to the conditions in those GITs. Bacterial genera that are acid-tolerant were uniquely identified in the GIT of pigs. This would make pig manures a suitable feedstock for biogas production process stabilisation when pH drops, and digester souring threatens. The identification of unique genera in the analysed biological replicates was explained by the different diets upon which the animals were being fed. Horse manure samples from replicates feeding on a purely plant-based diet had cellulolytic genera unique to it while those replicates feeding on a diet with a complex nutritional mix such as whey had specific genera unique to them. In biogas production systems, the genera identified in horse and cow manures would enhance the degradation of the cellulolytic substrate to produce biogas more efficiently since they have the ability to break down cellulolytic material. The ammonia tolerant groups also identified in pig manures would help reduce the risk of ammonia inhibition in systems prone to such inhibition thus increasing the AD process stability. Free-range cows are plausibly less likely to suffer rumen acidosis due to a natural selection of feeding on a variety of plant-based material over-feeding on refined feeds and therefore can maintain optimally neutral pH conditions which favour certain genera that are not acid-tolerant thus uniquely identified in their manure. This gives free-range cow manures a doubled positive impact on biogas production processes since they both have both cellulose degradation and acid tolerance capabilities. Chicken manure replicate samples collected from a source where the chickens shared the same space as cows e.g., roadrunners and free-range cows showed unique genera not identified in other chicken manures due to the ingestion of genera resident in some cow manure as the chickens eat bugs, worms, undigested feed, etc. Having observed the similarities and differences among the bacterial genera from cow, chicken, horse, and pig manures and the factors influencing the observations, the study revealed the relevance of the observed genera to biogas production, with pig manure having the highest diversity of identified genera, followed by cow, then horse and finally chicken manures. This insight on the varying diversities opens a door to further empirical investigation of their effects on biogas quantity and quality. This makes routine preliminary analysis of manure intended for anaerobic digestion a useful inclusion in the upscale process.

## 6. Recommendations

The taxonomy and diversity of microbial communities resident in cow, chicken, horse, and pig manures and factors leading to their presence have been elucidated. This paves the way for further investigations into the metagenomic functions of these taxa, determination of biological pathways followed by each community of genera, and possible identification of strategies to boost the production of methane using these biological catalysts. One approach is to use the metagenomic data, not only for predictive functioning but also an exploratory investigation of the various metabolites linked to these successional pathways (metabolomics) to provide information that hopefully expands our understanding of the roles of the microorganisms in their synergistic interspecific interactions during the degradation processes that yields biogas thus, providing insights useful for process optimisation.

## Figures and Tables

**Table 1:** Animal Manure Samples and Sources.

Sample	Sample ID	Sources	Location	Description of Farm (Location) Where Samples Were Collected from
Cow dung	C1	Boerdery Farm Produce	−26.21779, 27.6414784	An animal feeds producing and cattle rearing farm in Randfontein, Gauteng, South Africa, that feeds livestock on grains and concentrates
	C2	Kates Farm	−26.0990952, 27.9103166	A bed and breakfast facility in the periphery of Johannesburg, Gauteng serving free-range chicken and beef
	C3	Bosheuvel Country Estates	−26.0249182, 27.8929324	A multi-purpose farm in the Muldersdrift, Johannesburg with an on-site vintage hotel, restaurant, event venues, and conference rooms where Pinzgauer cattle, chickens, sheep, and pigs fed mostly on mixed vegetables and tubers are reared and served to guests
Chicken droppings	F1	Kates Farm	−26.0990952, 27.9103166	A bed and breakfast facility in the periphery of Johannesburg, Gauteng serving free-range chicken and beef
	F2	Country Portion Farm	−26.224517, 27.6325603	A farm in Randfontein that produces vegetables, poultry, and pork products to supply local restaurants and consumers. The poultry and pigs are fed on corn and sorghum and nutrient concentrates
	F3	Bosheuvel Country Estates	−26.0249182, 27.8929324	A multi-purpose farm in the Muldersdrift, Johannesburg with an on-site vintage hotel, restaurant, event venues, and conference rooms where Pinzgauer cattle, chickens, sheep, and pigs fed mostly on mixed vegetables and tubers are reared and served to guests
Horse manure	H1	Earth Centre	−26.080926, 27.8747353	A non-profit making organisation in Ruimsig, Gauteng that caters to disabilities such as Autism, ADD / ADHD, Cerebral Palsy, Downs Syndrome, and the Deaf using locally reared horses in Equine Assisted Programmes. The horses are fed with a wide range of feed concentrates and probiotics. They also feed on hay where they sleep.
	H2	Harveston Stables	−26.0990952, 27.9103167	A horse riding facility in Honeydew, Gauteng that offers lessons, pony rides, stabling, kids’ pony parties, and picnics. The horses are fed mainly on feed concentrates and they gave grass as their bedding.
	H3	Barent Horse Stables	−26.21779, 27.6414783	A plot rearing horses for family leisure in Randfontein, Gauteng. The horses are fed mostly on lucerne
Pig manure	P1	Bosheuvel Country Estates	−26.0249182, 27.8929324	A multi-purpose farm in the Muldersdrift, Johannesburg with an on-site vintage hotel, restaurant, event venues, and conference rooms where Pinzgauer cattle, chickens, sheep, and pigs fed mostly on mixed vegetables and tubers are reared and served to guests
	P2	Bosheuvel Country Estates	−26.0249182, 27.8929324	
	P3	Country Portion Farm	−26.224517, 27.6325603	A farm in Randfontein that produces vegetables, poultry, and pork products to supply local restaurants and consumers. The poultry and pigs are fed on corn and sorghum and nutrient concentrates

**Table 2:** Elemental Nutrient Utilisation by Various Animal Species.

Sample ID	C-Utilisation Rate (%)	H-Utilisation Rate (%)	N-Utilisation Rate (%)	S-Utilisation Rate (%)
C1	33.5 ± 0.3	36.9 ± 0.13	7.96 ± 0.08	38.9 ± 0.02
C3	35.1 ± 0.3	31.3 ± 0.12	6.20 ± 0.06	35.8 ± 0.02
F2	4.52 ± 0.35	47.6 ± 0.13	5.63 ± 0.09	13.2 ± 0.02
F3	7.43 ± 0.2	46.4 ± 0.11	5.06 ± 0.06	18.0 ± 0.02
P1	13.3 ± 0.2	5.86 ± 0.12	1.31 ± 0.05	40.1 ± 0.02
P2	12.8 ± 0.3	9.53 ± 0.12	0.928 ± 0.05	52.1 ± 0.02
P3	9.36 ± 0.4	6.92 ± 0.18	2.44 ± 0.07	47.4 ± 0.01
H1	17.8 ± 0.4	15.2 ± 0.12	43.1 ± 0.04	21.7 ± 0.02
H2	15.4 ± 0.3	13.6 ± 0.11	41.4 ± 0.04	19.9 ± 0.02
H3	15.0 ± 0.3	13.9 ± 0.12	41.2 ± 0.05	21.0 ± 0.02

Note: C2 and F1 were to be found in a free-range feeding scheme and therefore sample collection and analysis were not feasible. Utilisation rate (%) also called Apparent digestibility (%) = 100 * (Nutrient intake in feed−Nutrient loss in faeces)/Nutrient intake.

**Table 3:** Estimated OTU Richness and Diversity Indices of the Animal Manure Samples Collected from different sources around Johannesburg, South Africa.

**Variable**	**C1**	**C2**	**C3**	**F1**	**F2**	**F3**
Valid reads	12,310	10,110	9469	12,263		19,320
OTUs	1316	408	1475	352	103	1255
Simpson_1-D	0.94	0.89		0.94	0.95	0.88
Shannon_H	4.90	3.17	6.07	3.72	3.94	4.08
Chao_1	1331.05	419.13	1529.51	432.29	188.55	1274.85
ACE	1377.64	442.42	1609.21	458.76	198.70	1330.69
	**P1**	**P2**	**P3**	**H1**	**H2**	**H3**
Valid reads	55,482	30,943	17,585	5152	71,805	17,500
OTUs	1628	941	234	971	333	339
Simpson_1-D	0.88	0.89	0.30		0.30	0.88
Shannon_H	3.62	3.42	1.07	5.57	1.00	2.80
Chao_1	1643.50	951.90	297.87	979.26	365.88	344.01
ACE	1689.24	983.54	318.43	1010.30	390.47	360.15

## Data Availability

The authors confirm that the data supporting the findings of this study are available within the article. The raw genomic sequence data used was generated at UNISA in CAES and will be uploaded to an appropriate repository but is however currently available from the corresponding author on request.

## Appendix A

**Table A1:** Elemental composition of animal feeds.

Animal	Location	Sample ID	Feed Type	%C	%H	%N	%S	%O
Cow	Boerdery Farm	C1	Cattle feed	42.7 ± 0.2	6.40 ± 0.1	4.47 ± 0.03	0.145 ± 0.01	46.3 ± 0.1
Chicken	Country Portion Farm	F2	Chicken feed	42.2 ± 0.2	6.12 ± 0.1	2.31 ± 0.05	0	49.4 ± 0.2
			Layers’ feed	38.9 ± 0.1	5.74 ± 0.1	2.53 ± 0.05	0.222 ± 0.01	52.6 ± 0.2
Pig		P3	Bran and white maize meal	38.3 ± 0.2	5.82 ± 0.1	1.72 ± 0.05	0.155 ± 0.01	54.0 ± 0.2
			Crushed yellow maize	39.9 ± 0.2	6.03 ± 0.2	1.42 ± 0.05	0	52.6 ± 0.2
Horse	EARTH Centre	H1	Equus Nice and Easy	43.0 ± 0.1	6.34 ± 0.1	3.36 ± 0.03	0.167 ± 0.01	47.1 ± 0.1
			Eragrostis horse feed	47.0 ± 0.3	6.06 ± 0.1	0.928 ± 0.01	0.171 ± 0.01	45.8 ± 0.1
			Active bacteria	36.6 ± 0.2	5.21 ± 0.1	2.68 ± 0.03	2.25 ± 0.02	53.2 ± 0.2
			Equus all-time balancer	39.1 ± 0.3	5.79 ± 0.2	3.20 ± 0.01	0.337 ± 0.01	51.6 ± 0.2
			Equus Train and Leisure	40.1 ± 0.2	5.93 ± 0.1	2.33 ± 0.03	0.249 ± 0.01	51.4 ± 0.2
Horse	Harveston Stables	H2	Horse feed	42.5 ± 0.2	6.08 ± 0.1	2.16 ± 0.02	0.220 ± 0.01	49.0 ± 0.2
Cow	Bosheuvel Country Estates	C3	Mixed vegetables	30.7 ± 0.1	4.53 ± 0.1	3.53 ± 0.03	0.488 ± 0.01	60.7 ± 0.2
Chicken		F3						
Pig		P1, P2						

**Table A2:** Elemental composition of animal manures.

Manure Type	Location	Sample ID	%C	%H	%N	%S	%O
Cow dung	Boerdery Farm	C1	28.4 ± 0.1	4.04 ± 0.03	5.72 ± 0.05	0.223 ± 0.01	61.6 ± 0.3
Cow dung	Kates Farm	C2	38.5 ± 0.1	5.32 ± 0.03	3.74 ± 0.03	0.217 ± 0.01	52.2 ± 0.3
Cow dung	Bosheuvel Country Estates	C3	20.0 ± 0.2	3.56 ± 0.02	7.17 ± 0.03	0.313 ± 0.01	69.0 ± 0.2
Chicken manure	Kates Farm	F1	38.8 ± 0.1	5.35 ± 0.04	1.75 ± 0.02	0.219 ± 0.01	53.9 ± 0.3
Chicken manure	Country Portion Farm	F2	23.7 ± 0.2	3.11 ± 0.03	9.80 ± 0.04	0.251 ± 0.01	63.1 ± 0.2
Chicken manure	Bosheuvel Country Estates	F3	33.0 ± 0.1	4.82 ± 0.01	1.69 ± 0.03	0.205 ± 0.01	57.3 ± 0.2
Pig manure	Bosheuvel Country Estates	P1	26.7 ± 0.1	4.26 ± 0.02	2.67 ± 0.02	0.292 ± 0.01	66.1 ± 0.2
Pig manure	Bosheuvel Country Estates	P2	29.9 ± 0.2	4.96 ± 0.02	3.63 ± 0.02	0.234 ± 0.01	61.3 ± 0.3
Pig manure	Country Portion Farm	P3	42.8 ± 0.2	5.52 ± 0.03	1.34 ± 0.02	0.271 ± 0.01	50.1 ± 0.2
Horse manure	EARTH Centre	H1	38.0 ± 0.2	5.56 ± 0.02	2.17 ± 0.02	0.313 ± 0.01	54.0 ± 0.2
Horse manure	Harveston Stables	H2	40.2 ± 0.1	5.25 ± 0.01	1.26 ± 0.02	0.176 ± 0.01	53.1 ± 0.2
Horse manure	Barent Horse Stables	H3	27.7 ± 0.2	3.63 ± 0.02	1.85 ± 0.03	0.219 ± 0.01	66.6 ± 0.3

## Appendix B

**Table A3:** Genera prevalent in various manure samples from various sources.

Cow Dung			
Genera Common in All Manure Samples	Uniquely Prevalent Genera Specific to Locations		
C1, C2, C3	Boerdery Farm Produce (C1)	Kates Farm (C2)	Bosheuvel Country Estates (C3)
*Lachnospiracaeae_uc* *Ruminoccocaceae_uc* *Sporobacter* *Christensenellaceae_uc* *Treponema* *Oscillibacter* *Terrisporobacter* *Turicibacter* *Clostridium* *Romboutsia* *Acinetobacter* *Corynebacterium*	*Glutamicibacter* *Enterococcus* *Jeotgalicoccus*	*Prevotella* *Lactobacillaceae_uc*	*Cellulosilyticum* *Enterobacter* *Lysinibacillus* *Psychrobacter*
Chicken droppings			
F1, F2, F3	Kates Farm (F1)	Country Portion Farm (F2)	Bosheuvel Country Estates (F3)
*Clostridium* *Acinetobacter* *Pseudomonas* *Lactobacillus*	*Oceanisphaera* *Glutamicibacter* *Escherichia* *Enterococcus* *Jeotgalicoccus*	*Oscillibacter* *Streptococcus*	*Christensenellaceae_uc* *Sporobacter*
Horse manure			
H1, H2, H3	Earth Centre (H1)	Harveston Stables (H2)	Barent Horse Stables (H3)
*Romboutsia* *Acinetobacter* *Pseudomonas* *Lactobacillus* *Corynebacterium* *Aerococcus* *Lactobacillaceae_uc* *Escherichia* *Enterococcus*	*Prevotella* *Christensenellaceae_uc* *Treponema*	*Lachnospiracaeae_uc* *Sporobacter* *Alistipes* *Oceanisphaera*	*Enterobacter* *Weissella* *Kosakonia*
Pig manure			
P1, P2, P3	Bosheuvel Country Estates (P1)	Bosheuvel Country Estates (P2)	Country Portion Farm (P3)
*Prevotella* *Cellulosilyticum* *Peptostreptococaceae_uc* *Clostridiaceae_uc* *Lachnospiracaeae_uc* *Ruminoccocaceae_uc* *Sporobacter* *Christensenellaceae_uc* *Treponema* *Ruminococcus* *Oscillibacter* *Terrisporobacter* *Turicibacter* *Clostridium* *Romboutsia* *Pseudomonas* *Streptococcus* *Lactobacillus* *Corynebacterium*	*Acinetobacter* *Lactobacillaceae_uc* *Enterococcus*	**---**	*Enterobacter* *Escherichia*

**Table A4:** Bacterial genera relevant for biogas production.

Cow Dung	Chicken Droppings	Horse Manure	Pig Manure
*Lachnospiracaeae_uc*	*Clostridium*	*Romboutsia*	*Prevotella*
*Ruminoccocaceae_uc*	*Lactobacillus*	*Lactobacillus*	*Cellulosilyticum*
*Sporobacter*	*Escherichia*	*Aerococcus*	*Peptostreptococaceae_uc*
*Christensenellaceae_uc*	*Enterococcus*	*Lactobacillaceae_uc*	*Clostridiaceae_uc*
*Treponema*	*Jeotgalicoccus*	*Escherichia*	*Lachnospiracaeae_uc*
*Oscillibacter*	*Oscillibacter*	*Enterococcus*	*Ruminoccocaceae_uc*
*Terrisporobacter*	*Streptococcus*	*Prevotella*	*Sporobacter*
*Turicibacter*	*Christensenellaceae_uc*	*Christensenellaceae_uc*	*Christensenellaceae_uc*
*Clostridium*	*Sporobacter*	*Treponema*	*Treponema*
*Romboutsia*		*Lachnospiracaeae_uc*	*Ruminococcus*
*Enterococcus*		*Sporobacter*	*Oscillibacter*
*Jeotgalicoccus*		*Alistipes*	*Terrisporobacter*
*Prevotella*		*Weissella*	*Turicibacter*
*Lactobacillaceae_uc*			*Clostridium*
*Cellulosilyticum*			*Romboutsia*
			*Streptococcus*
			*Lactobacillus*
			*Lactobacillaceae_uc*
			*Enterococcus*
			*Escherichia*
